# Dietary habits of polar bears in Foxe Basin, Canada: possible evidence of a trophic regime shift mediated by a new top predator

**DOI:** 10.1002/ece3.2173

**Published:** 2016-07-28

**Authors:** Melissa P. Galicia, Gregory W. Thiemann, Markus G. Dyck, Steven H. Ferguson, Jeff W. Higdon

**Affiliations:** ^1^ Department of BiologyYork UniversityTorontoOntarioM3J 1P3Canada; ^2^ Faculty of Environmental StudiesYork UniversityTorontoOntarioM3J 1P3Canada; ^3^ Wildlife Research SectionDepartment of EnvironmentGovernment of NunavutP.O. Box 209IgloolikNunavutX0A 0L0Canada; ^4^ Fisheries and Oceans Canada501 University CrescentWinnipegManitobaR3T 2N6Canada; ^5^ Higdon Wildlife Consulting912 Ashburn StreetWinnipegManitobaR3G 3C9Canada

**Keywords:** Bowhead whales (*Balaena mysticetus*), Canadian Arctic, climate change, feeding ecology, killer whales (*Orcinus orca*), marine food web, marine mammals, polar bear (*Ursus maritimus*), quantitative fatty acid signature analysis

## Abstract

Polar bear (*Ursus maritimus*) subpopulations in several areas with seasonal sea ice regimes have shown declines in body condition, reproductive rates, or abundance as a result of declining sea ice habitat. In the Foxe Basin region of Nunavut, Canada, the size of the polar bear subpopulation has remained largely stable over the past 20 years, despite concurrent declines in sea ice habitat. We used fatty acid analysis to examine polar bear feeding habits in Foxe Basin and thus potentially identify ecological factors contributing to population stability. Adipose tissue samples were collected from 103 polar bears harvested during 2010–2012. Polar bear diet composition varied spatially within the region with ringed seal (*Pusa hispida*) comprising the primary prey in northern and southern Foxe Basin, whereas polar bears in Hudson Strait consumed equal proportions of ringed seal and harp seal (*Pagophilus groenlandicus*). Walrus (*Odobenus rosmarus*) consumption was highest in northern Foxe Basin, a trend driven by the ability of adult male bears to capture large‐bodied prey. Importantly, bowhead whale (*Balaena mysticetus*) contributed to polar bear diets in all areas and all age and sex classes. Bowhead carcasses resulting from killer whale (*Orcinus orca*) predation and subsistence harvest potentially provide an important supplementary food source for polar bears during the ice‐free period. Our results suggest that the increasing abundance of killer whales and bowhead whales in the region could be indirectly contributing to improved polar bear foraging success despite declining sea ice habitat. However, this indirect interaction between top predators may be temporary if continued sea ice declines eventually severely limit on‐ice feeding opportunities for polar bears.

## Introduction

Ecological flexibility can play an important role in a species’ ability to cope with environmental change. In contrast, highly specialized species can be particularly sensitive to climate change and habitat loss (Colles et al. 2009; Kovacs et al. 2011; Gilg et al. 2012). Polar bears (*Ursus maritimus*) are top predators within their Arctic circumpolar range and may be sensitive to environmental change because of their reliance on sea ice habitat for traveling, mating, and foraging (Stirling and Derocher 1993; Laidre et al. 2008). As sea ice extent and habitat quality decline because of climate warming, ice‐associated prey species become less accessible. For instance, capture‐based research on polar bears in Hudson Bay and the Southern Beaufort Sea has documented habitat‐mediated nutritional stress (Amstrup et al. 2006; Stirling et al. 2008) and ultimately reduced body condition, reproductive rates, survival, or abundance (Stirling et al. 1999; Obbard et al. 2006; Regehr et al. 2007; Rode et al. 2010, 2014; Bromaghin et al. 2015). The effects of environmental changes on polar bears in other parts of their range, where capture‐based research is rare, are poorly understood.

Across their circumpolar range, polar bears feed primarily on ringed seals (*Pusa hispida*) and bearded seals (*Erignathus barbatus;* Stirling and Archibald 1977; Smith 1980; Stirling and Øritsland 1995; Thiemann et al. 2008a). Locally available marine mammals, including harp seals (*Pagophilus groenlandicus*) and beluga whales (*Delphinapterus leucas*), are also important prey for polar bears in some parts of their range (e.g., Thiemann et al. 2008a; Galicia et al. 2015). In Alaska, polar bears have been observed scavenging the carcasses of bowhead whales (*Balaena mysticetus*) left on shore during subsistence harvests (Miller et al. 2006; Bentzen et al. 2007; Schliebe et al. 2008; Herreman and Peacock 2013). In Foxe Basin, polar bear diets are thought to be dominated by ringed seal, with comparatively minor contributions from harp, harbor (*Phoca vitulina*), and bearded seal (Thiemann et al. 2008a). However, little is known about spatial patterns of foraging within Foxe Basin as previous studies only considered the mean diet of bears in the 1.18 million km^2^ region, which includes areas with diverse ecological conditions and potentially varying prey.

Foxe Basin is seasonally ice‐free, and polar bears are forced to migrate to shore and rely on stored fat for energy when the sea ice melts each summer. The sea ice season has declined from 9 to 7 months since 1979 (Sahanatien and Derocher 2012). As a result, the polar bear fasting period begins earlier and lasts longer, reducing the foraging time available to accumulate fat in the spring and early summer (Stirling and Parkinson 2006; Sahanatien and Derocher 2012). Increased sea ice fragmentation (Sahanatien and Derocher 2012) may reduce the availability of preferred prey and increase the energetic cost of foraging (Stirling et al. 1999; Regehr et al. 2007; Rode et al. 2012; Sahanatien and Derocher 2012). Nevertheless, the size of the Foxe Basin subpopulation has remained stable since the mid‐1990s under a sustainable harvest regime with an estimated population size of 2585 (CI 95%: 2096–3189) in 2009–2010 (Stapleton et al. 2016). The ecological factors supporting this stable subpopulation in the face of declining sea ice quality and duration are unclear.

Declining sea ice has created the potential for a shift in food web dynamics in Foxe Basin, as killer whales (*Orcinus orca*) have expanded their range into the region, where they feed on bowhead whales, ringed seals, beluga whales, and narwhals (*Monodon monoceros*; Higdon and Ferguson 2009; Higdon et al. 2012). The Eastern Canada–West Greenland (EC‐WG) bowhead whale population was over‐harvested from the 1500s through to the early 1900s with over 70,000 animals taken by commercial whalers and in subsistence hunts (Higdon 2010). With the end of commercial harvesting ca. 1915 and a small co‐managed subsistence harvest starting in 1996, the bowhead whale population has since increased to an estimated 7660 whales (95% HDI 4500–11,000; Frasier et al. 2015). Northern Foxe Basin serves as an important summer nursery and feeding ground for bowhead cow–calf pairs and juveniles, where whales may be protected in spring by hundreds of kilometers of heavy pack ice (Cosens and Blouw 2003; Higdon et al. 2012). Killer whales tend to target smaller (i.e., younger) individuals (Ford and Reeves 2008; Ferguson et al. 2012b), which subsequently increases the vulnerability of calves and juveniles to killer whale predation in northern Foxe Basin in a reduced sea ice habitat (Higdon et al. 2012). The preference of killer whales to feed on the head and mouthparts of a baleen whale (Jefferson et al. 1991; Ford et al. 2005) generates a large carcass that can drift on shore, potentially creating an important supplementary food source for polar bears.

Although dedicated research has yet to be carried out, the increasing bowhead whale population, expansion of killer whales’ range, and declining sea ice suggest the possibility of an ongoing ecological regime shift in Foxe Basin. A near‐term consequence of a shift toward a more temperate food web would be a change in the ecological role of polar bears, including altered diet composition and an increase in scavenging on the prey of killer whales. Our objective was to characterize the diets of polar bears in Foxe Basin over regional and local spatial scales. We hypothesized that polar bear diet composition within Foxe Basin would vary spatially as a result of variable ecological conditions. We also hypothesized that bowhead whale carcasses may be providing an additional food source for polar bears during the ice‐free season as a result of natural mortality, killer whale predation, and to a lesser extent, anthropogenic mortality (including harvest, struck and loss, net entanglement, and ship collisions; DFO 2015).

## Materials and Methods

### Sample collection

**Figure 1:**
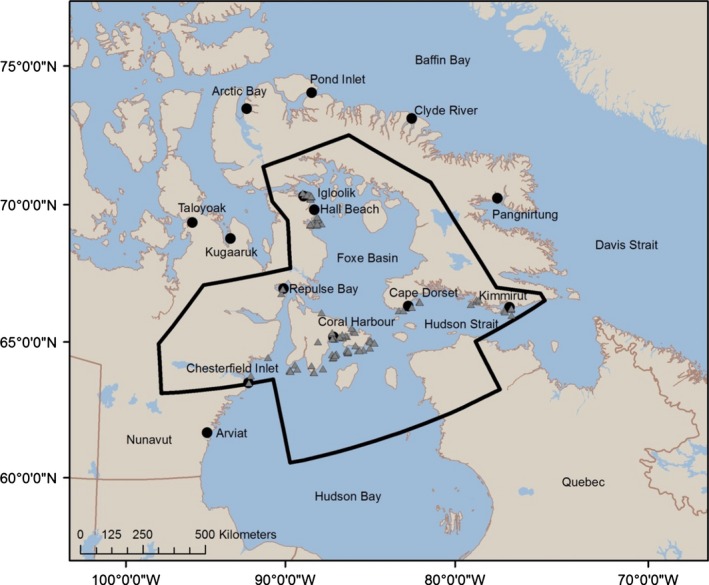
Location of polar bears (*n* = 103) harvested by local Inuit hunters during 2010–2012 across the Foxe Basin subpopulation (solid line; Obbard et al. 2010). Harvest locations of polar bears are represented as (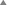). Communities where marine mammals (*n* = 202) were collected for this study from 2003 to 2012 are represented as (●).

We examined adipose tissue samples from 103 individual polar bears (Table 1) harvested across the Foxe Basin subpopulation (Fig. 1). Tissue samples were collected by Inuit hunters during the course of annual subsistence hunts from July 2010 to June 2012. Samples were collected from male and female bears and across age classes: adults (5+ years old), subadults (3–4 years old), and independent 2‐year‐olds. Management targets a 2:1 male:female sex ratio in the harvest and prohibits taking females with dependent cubs. A sample of subcutaneous adipose tissue (ca. 6 cm × 3 cm) was taken from the rump of each bear and wrapped in aluminum foil, sealed in a labeled Whirl‐Pak and stored at −20°C until analysis.

**Table 1:** Distribution of adipose tissue samples collected from polar bears taken during the 2010/2011 and 2011/2012 subsistence harvest seasons across the Foxe Basin subpopulation

Region	Community	Total (*n*)	Adult		Subadult			Independent, 2 years old	
			F	M	F	M	Unk	F	M
Northern Foxe Basin	Hall Beach	14	1	4	5	3	1	0	0
	Igloolik	13	2	7	1	3	0	0	0
Eastern Foxe Basin	Cape Dorset	5	1	2	0	2	0	0	0
Hudson Strait	Kimmirut	12	2	8	1	1	0	0	0
Southern Foxe Basin	Chesterfield Inlet	3	0	2	0	1	0	0	0
	Coral Harbour	54	7	25	14	7	0	1	0
	Repulse Bay	2	1	1	0	0	0	0	0
	Total	103	14	49	21	17	1	1	0

We analyzed 243 marine mammal blubber samples from bearded seals (*n* = 43), beluga whales (*n* = 31), bowhead whales (*n* = 5), harbor seals (*n* = 17), harp seals (*n* = 9), narwhals (*n* = 37), ringed seals (*n* = 48), and Atlantic walrus (*n* = 17) harvested during annual subsistence hunts in Foxe Basin and adjacent polar bear subpopulation zones from 2003 to 2012 (Fig. 1). In addition, bowhead whale skin and outer blubber samples (*n* = 36) were collected in the Foxe Basin and Repulse Bay regions between July and August in 2008 and 2009 using a crossbow darting system (Pomerleau et al. 2014). The collection of walrus, seal, and whale samples included all sex and age classes. Samples were wrapped in aluminum foil and placed in a labeled Whirl‐Pak bag and stored at −20°C.

### Laboratory analysis

A subsample approximately 0.5 g was taken through the entire depth of each adipose tissue sample to avoid any oxidized surfaces. Lipid was quantitatively extracted according to Iverson et al. (2001). We used sulfuric acid as a catalyst to derive fatty acid methyl esters (FAME) from the extracted lipid (Budge et al. 2006). FAME were analyzed in duplicate using temperature‐programmed gas chromatography on a Perkin Elmer Autosystem II capillary gas chromatograph (GC) with a flame ionization detector (FID), using a polar column (Agilent Technologies, DB‐23; 30 m × 0.25 mm ID; Budge et al. 2006). Typically, over 70 FAs are identified in each adipose tissue sample and expressed as the mass percentage of the total FA ± 1 SEM. FAs are identified using the nomenclature A:Bn‐X, where A is the carbon chain length, B is the number of double bonds, and X is the position of the first double bond in relation to the terminal methyl group.

### QFASA modeling

We used quantitative fatty acid signature analysis (QFASA; Iverson et al. 2004) to estimate the proportional biomass of each prey species in the diet of polar bears. The QFASA model compares the average prey FA profile (or “signature”) with each individual predator FA profile and determines the weighted combination of prey FA that minimizes the Kullback–Leibler distance to the predator's FA signature, after accounting for patterns of FA metabolism (Iverson et al. 2004; Budge et al. 2006). We used calibration coefficients derived from captive mink (*Neovison vison*) raised on a controlled marine‐based diet (Thiemann et al. 2008a) to account for the fact that FA may be modified, utilized, or biosynthesized before deposition in the predator's adipose tissue (Iverson et al. 2004). QFASA‐based diet estimates reflect the integrated feeding habits of an individual predator over the preceding weeks to months, on a lipid biomass basis (Iverson et al. 2004; Budge et al. 2011).

Polar bear diets were estimated using 30 FA obtained solely or primarily from diet (Iverson et al. 2004). Our FA set was similar to that used in previous polar bear diet studies (Thiemann et al. 2008a), except for the exclusion of 20:1*n*‐11 as it appeared to contribute to overlap among prey FA profiles in simulated diet estimates (Galicia et al. 2015). Our dataset included eight ecologically relevant and accessible prey species for polar bears in Foxe Basin. We used diet simulations to assess the ability of the QFASA model to accurately distinguish among prey types based on their FA signature (Appendix S1). Diet simulations indicated that spatial variability within a prey species was small relative to variability among species (see also Thiemann et al. 2008a,b), and thus, prey species from different regions were pooled together. All diet simulations and QFASA estimates were performed in R (R Version 2.1.0, The R Foundation for Statistical Computing, 2005).

### Statistical analyses

Intrapopulation differences in polar bear diet composition were analyzed using randomization–permutation MANOVA because diet data were not normally distributed (Anderson 2001a,b). We used two‐way MANOVA to identify potential spatial differences within the subpopulation while controlling for sex effects. Two‐way MANOVA was used to test sex and age class differences within each geographic area. We also tested for seasonal variation in the diet composition of polar bears in each geographic area (one‐way MANOVA) separately. We did not control for sex variation because there were too few samples collected from each sex in every season. Seasons were defined as fall (September–November), winter (December–February), spring (March–May), and summer (June‐August). Seasonal patterns in polar bear foraging were based on the timing of sample collection. Because fat stores reflect integrated diet composition over the preceding weeks to months (Iverson et al. 2004), there is some lag between the ingestion of prey and its detectability in the fat stores of a predator. Nevertheless, the assimilation of dietary fatty acids is rapid enough to make inferences about dietary patterns across broad temporal (i.e., seasonal) scales (Nordstrom et al. 2008; Thiemann et al. 2008a). All statistical tests were performed in R (R Version 2.15.3, The R Foundation for Statistical Computing, 2013).

## Results

### Polar bear diet composition

#### Spatial variation in polar bear diet

**Figure 2:**
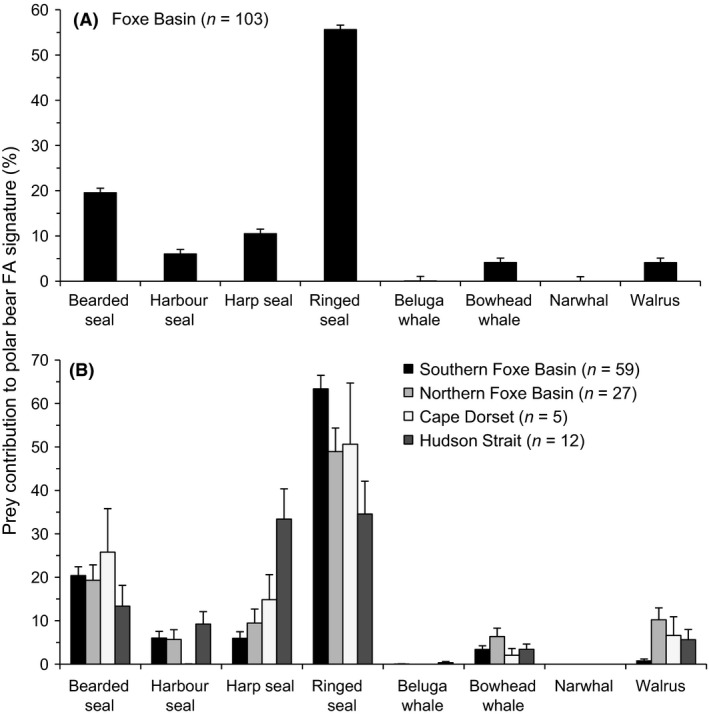
Diet composition of polar bears (A) pooled across the Foxe Basin subpopulation (*n* = 103) and (B) separated by region (eastern Foxe Basin: Cape Dorset; Hudson Strait: Kimmirut; northern Foxe Basin: Hall Beach and Igloolik; southern Foxe Basin: Chesterfield Inlet, Coral Harbour, and Repulse Bay) during 2010–2012. Data represent each prey species’ biomass contribution to polar bear diet estimates, expressed as mean ± SE.

Ringed seal was the dominant prey species in Foxe Basin polar bear diets (mean ± SEM: 56 ± 2.7%), followed by bearded seal (20 ± 1.6%) and harp seal (11 ± 1.7%; Fig. 2A). Harbor seal, bowhead whale, and walrus were minor dietary components comprising 6 ± 1.1%, 4 ± 0.7%, and 4 ± 0.9%, respectively, across the entire subpopulation. Four main clusters of samples were used to examine regional differences: northern (Hall Beach and Igloolik), southern (Coral Harbour, Chesterfield Inlet, and Repulse Bay), eastern (Cape Dorset), and Hudson Strait (Kimmirut).

The diets of bears in northern Foxe Basin, southern Foxe Basin, and Hudson Strait were all significantly different from each other (permutation two‐way MANOVA, *P* < 0.030; Fig. 2B). However, diet composition around Cape Dorset was not significantly different from any of the other areas (permutation two‐way MANOVA, *P* ≥ 0.170 in all cases), likely because of small sample size (*n* = 5). Cape Dorset could not be reasonably pooled with any of the three other geographic clusters (northern Foxe Basin, southern Foxe Basin, and Hudson Strait) and thus was excluded from further analysis.

Bearded seal and harbor seal consumption did not vary across the study area (permutation ANOVA, *P* = 0.395 and *P* = 0.663, respectively). Bearded seal was the second most consumed prey species in southern Foxe Basin (20 ± 2.0%) and northern Foxe Basin (19 ± 3.5%). Ringed seal consumption was highest in southern Foxe Basin (63 ± 3.1%) and northern Foxe Basin (49 ± 5.4%) compared to Hudson Strait (permutation ANOVA, *P* < 0.001). In contrast, bears in Hudson Strait consumed roughly equal proportions of ringed seal and harp seal (35 ± 7.6% and 33 ± 6.9%, respectively). Harp seal was more abundant in Hudson Strait diets than either southern Foxe Basin or northern Foxe Basin (permutation ANOVA, *P* < 0.001). In southern and northern Foxe Basin, ringed seal was present in the diet of the majority of bears (98% and 89% of bears, respectively), whereas harp seal was less frequent (46% and 44% of bears, respectively). In contrast, ringed seal and harp seal were found at equal frequencies in Hudson Strait bears (both prey species present in 75% of bears).

There was no significant spatial difference in the level of bowhead whale consumption (permutation ANOVA, *P* = 0.262), although bowhead was consumed most frequently by bears in northern Foxe Basin (56% of bears), followed by Hudson Strait (50% of bears) and southern Foxe Basin (37% of bears). There was no detectable narwhal consumption by any individual and beluga whale biomass was found in low levels (<3%) in 3% of southern Foxe Basin bears and 17% of Hudson Strait bears. No bears in northern Foxe Basin had detectable levels of beluga whale. Walrus consumption was significantly higher in northern Foxe Basin and Hudson Strait than southern Foxe Basin (permutation ANOVA, *P* < 0.001). Walrus consumption was also more frequent in northern Foxe Basin and Hudson Strait (63% and 67% of bears, respectively), and relatively rare in southern Foxe Basin (14% of bears).

#### Age‐ and sex‐specific variation in polar bear diet

**Figure 3:**
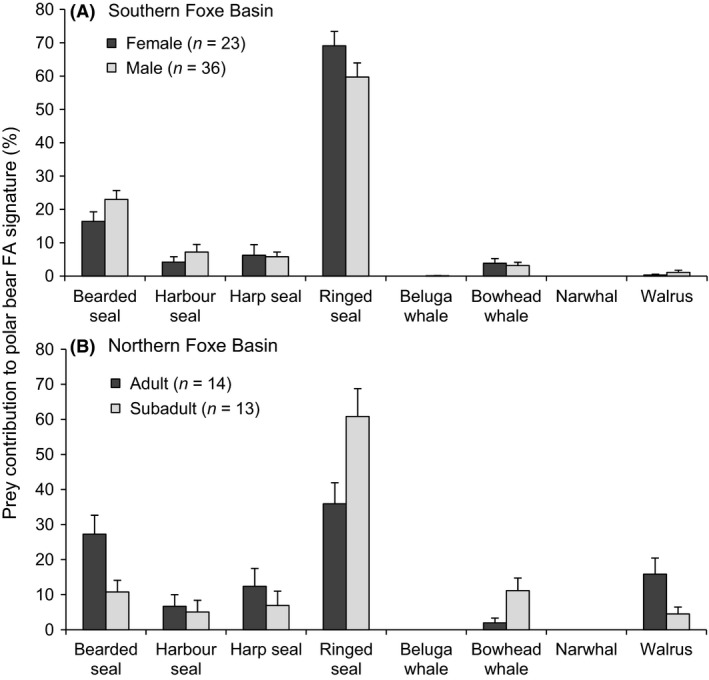
Sex and age class variation in diets of polar bears in (A) southern Foxe Basin and (B) northern Foxe Basin during 2010–2012. Diet estimates are represented as mean ± SE.

In southern Foxe Basin, there was a significant sex‐specific difference in overall diet composition (permutation two‐way MANOVA, *P* = 0.040; Fig. 3A) and no significant difference across age class (permutation two‐way MANOVA, *P* = 0.139). However, consumption of particular prey species did not significantly differ between female and male bears (permutation *t*‐test, *P* ≥ 0.091 in all cases). Ringed seal was the dominant prey in all age classes and sexes and was consumed by nearly all bears (96% of female bears and 100% of male bears). Bearded seal was also present in the large majority of bears (91% of female bears and 97% of male bears).

Diets of bears in northern Foxe Basin were significantly influenced by age class but not sex (permutation two‐way MANOVA, *P* = 0.027, Fig. 3B and *P* = 0.133, respectively). Ringed seal consumption was higher in subadult bears (61 ± 8.0%) than adults (36 ± 6.0%; permutation *t*‐test, *P* = 0.022), whereas adult bears consumed more bearded seal than did subadults (27 ± 5.4% and 12 ± 3.5%, respectively, permutation *t*‐test, *P* = 0.031). The higher level of bearded seal and lower level of ringed seal in adult bears was primarily driven by adult males. Bowhead whale consumption was significantly higher in subadult bears than in adults (11 ± 3.6% and 2 ± 1.4%, respectively; permutation *t*‐test, *P* = 0.022). Bowhead whale was also consumed more frequently in subadults (77% of bears) compared to adults (36% of bears). Walrus consumption was the highest in adult bears (permutation *t*‐test, *P* = 0.045), which was driven by adult males with the highest contribution to the diets in northern Foxe Basin (18 ± 5.6% in adult males). Too few samples were collected in Hudson Strait for statistical analysis on the effects of age class and sex.

#### Seasonal variation in polar bear diet

**Figure 4:**
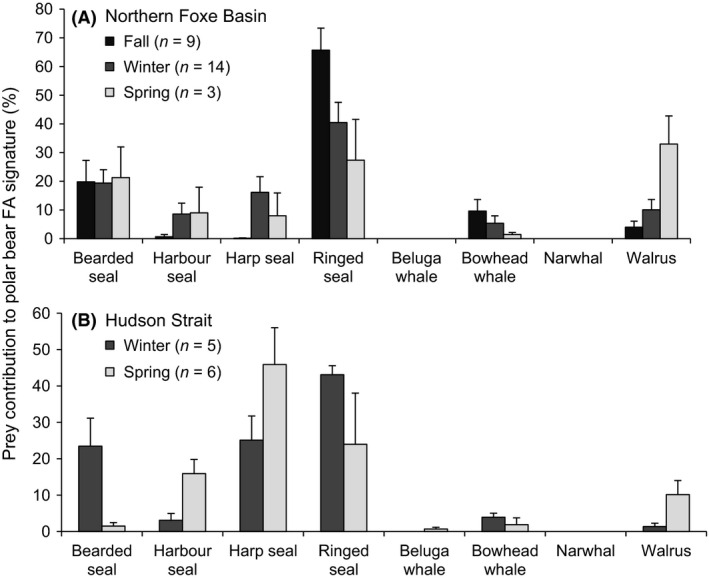
Seasonal diet composition of polar bears in (A) northern Foxe Basin and (B) Hudson Strait during 2010–2012. Diet estimates are represented as mean ± SE.

Polar bear diets differed seasonally in northern Foxe Basin (permutation MANOVA, *P* = 0.030; Fig. 4A) and Hudson Strait (permutation MANOVA, *P* = 0.044; Fig. 4B), but not in southern Foxe Basin (permutation MANOVA, *P* = 0.192). In northern Foxe Basin, summer was excluded from the analysis as there was only one sample collected in August. Based on the date of sampling, ringed seal consumption was highest in bears in the fall (permutation ANOVA, *P* = 0.015) and was found in 100% of bears sampled. Walrus consumption was highest in bears in the spring (33 ± 9.8%; permutation ANOVA, *P* = 0.006) and present in 100% of bears sampled.

In Hudson Strait, bearded seal consumption was higher (24 ± 7.7%) during the winter than spring (2 ± 1.0%; permutation *t*‐test, *P* = 0.030). Bearded seal consumption was also more frequent among bears in winter (80% of bears) than spring (33% of bears). Harbor seal consumption was higher during the spring (16 ± 3.9%) compared to winter (3 ± 1.9%; permutation *t*‐test, *P* = 0.035) and more frequent in spring (83% of bears) than winter (60% of bears). Harp seal consumption in the spring (46 ± 14%) was not significantly different than in winter (25 ± 6.7%, permutation *t*‐test, *P* = 0.160), likely because of limited statistical power. Walrus consumption was also not statistically different in spring (10 ± 3.9%) than winter (1 ± 0.9%; permutation *t*‐test, *P* = 0.069).

## Discussion

Our study was the first to identify spatial differences in polar bear diet composition in the Foxe Basin subpopulation and the first to quantify the contribution of bowhead whale to the diets of polar bears in the eastern Arctic. Our results suggest that polar bears in this region are exploiting locally available prey and may seasonally shift their foraging preferences. Given the apparently stable size of the Foxe Basin subpopulation (Stapleton et al. 2016), despite recent declines in habitat quality (Sahanatien and Derocher 2012), our results suggest the diversity of dietary alternatives to ringed and bearded seals may help buffer Foxe Basin polar bears from the effects of sea ice loss, at least in the early stages. Moreover, the presence of bowhead whale in the diets of bears suggests that scavenging carcasses provided by killer whale predation may serve as an important energetic supplement, particularly during seasons of low food availability and to younger age classes that may have difficulty catching prey. Our results provide a better understanding of polar bear feeding ecology in a region currently undergoing changes in sea ice habitat.

### Spatial variation in polar bear diet

Polar bears in Foxe Basin have access to a variety of potential prey, which is reflected in their diverse diet composition (Fig. 2). The primary prey of polar bears across the study area was ringed seal, a trend consistent with previous coarse‐scale analyses of the Foxe Basin food web (Thiemann et al. 2008a) and likely a consequence of the ubiquitous distribution and high abundance of ringed seals. Spatial differences in diet composition across Foxe Basin may reflect the importance of locally available prey or carrion (such as bowhead whales). Our study analyzed spatial variation by clustering polar bears into four main geographic groups, including Cape Dorset, where only five samples were available. The spatial patterns we identified may become clearer with increased sampling in the region. Sahanatien et al. (2015) also identified a similar intrapopulation spatial structure to the polar bear feeding clusters identified in our study; the three spatial clusters identified were influenced by changing sea ice dynamics in the Foxe Basin subpopulation.

Polar bears in southern Foxe Basin had the least diverse diet with the majority of the diet comprised of ringed seal and few alternate prey species (Fig. 2B). Polar bear diets in this southern area of Foxe Basin are similar to the diet composition of bears in Western Hudson Bay (Iverson et al. 2006; Thiemann et al. 2008a) suggesting a shared food source between adjacent subpopulations with partially overlapping utilization distributions (Sahanatien and Derocher 2010; McCall et al. 2015). The prominence of ringed seal in southern Foxe Basin polar bear diets may be a consequence of high densities of ringed seals attracted to the biological productivity of the recurring polynya in the area of Roes Welcome Sound (Stirling 1980; Stirling et al. 1981). Polar bears in northern Foxe Basin also consumed high levels of ringed seals, but had greater dietary diversity including bearded seal, harbor seal, harp seal, bowhead whale, and walrus (Fig. 2B).

Polar bears in Hudson Strait had the highest dietary diversity of all areas and consumed roughly equal proportions of ringed seal and harp seal (Fig. 2B). These patterns are more similar to the diets of polar bears in the adjacent Davis Strait subpopulation than other areas in Foxe Basin. A high consumption of harp seal was previously identified in Davis Strait polar bears (Iverson et al. 2006; Thiemann et al. 2008a). Our results suggest that polar bears in Hudson Strait may be moving eastward into Davis Strait to exploit harp seal which have increased in numbers over the past four decades (DFO 2011). In addition to the movement of bears, dietary similarities in adjacent subpopulations may be a function of shared, migratory prey. Harp seals commonly move from the Labrador Sea into and through Hudson Strait as the sea ice recedes in summer (Sergeant 1976). Given that climate related changes in sea ice are expected to alter the distribution and migratory patterns of polar bear prey (e.g., Bailleul et al. 2012; Chambellant et al. 2012), future research into polar bear diets could provide insights into warming‐related changes in Arctic marine ecosystems.

Three walrus stocks overlap the range of polar bears in Foxe Basin (Stewart et al. 2014). Little is known about the seasonal movements of walrus, however they are found in high concentrations year‐round in northern Foxe Basin, northwestern Hudson Bay, and Hudson Strait (DFO 2002, COSEWIC 2006). Although walrus overlap with polar bears throughout Foxe Basin, only northern Foxe Basin, Cape Dorset, and Hudson Strait polar bears seem to be making substantial use of this resource (Fig. 2B).

Bowhead whale was present in the diets of polar bears in all four regions of Foxe Basin; however consumption was highest in northern Foxe Basin followed by Hudson Strait. Bowhead whales are too large to be killed by polar bears, but carcasses become available from natural stranding/mortality, remains from subsistence hunts, anthropogenic mortality, and predation by killer whales. Carcasses represent an opportunistic food source that can provide a high caloric intake for some individuals (Miller et al. 2006; Higdon and Ferguson 2010; Herreman and Peacock 2013; Rode et al. 2014). For instance, high concentrations of polar bears have been observed scavenging on bowhead whale remains from subsistence harvests along the Alaskan coast throughout the fall and winter (Miller et al. 2006; Schliebe et al. 2008; Herreman and Peacock 2013; Rogers et al. 2015). Schliebe et al. (2008) recorded upwards of 65 polar bears on a single bowhead whale carcass. In 2009, Stapleton et al. (2016) observed 11 bears scavenging on a bowhead whale carcass in Foxe Basin. Larsen (1986) described the presence of 56 polar bears around a bowhead carcass floating in the drift ice near Svalbard and presented evidence that the whale may have attracted bears from a considerable distance (>100 km) away.

In Foxe Basin, the bowhead whale stock aggregates in two summer feeding areas: northwestern Hudson Bay around Repulse Bay and northern Foxe Basin near Igloolik Island (Cosens and Innes 2000; Cosens and Blouw 2003). Hudson Strait is a wintering ground for the majority of the EC‐WG bowhead whale population which remain in the dense pack ice (Koski et al. 2006; Ferguson et al. 2010). Northern Foxe Basin acts as a summer feeding ground for bowhead whale cow–calf pairs and juveniles, which are target prey of killer whales (Cosens and Blouw 2003; Ferguson et al. 2012b). In contrast to the situation in northern Alaska, where an average of 18 harvested bowhead whale carcasses may be available to polar bears (Herreman and Peacock 2013), total allowable harvest is five bowhead whale per year across Nunavut (DFO 2015). Since the end of commercial whaling (1918–2009), there have been a minimum total of 65 bowhead whales harvested from the EC‐WG population, including 14 whales that were struck and lost (Higdon 2010). There is evidence of polar bears scavenging on bowhead whale carcasses in Hall Beach (i.e., northern Foxe Basin) from subsistence harvest, however due to the limited number of bowhead whales landed (or struck and lost) per year, most carcasses likely arise from killer whale depredation events (NWMB 2000, Higdon and Ferguson 2010; Ferguson et al. 2012a).

**Figure 5:**
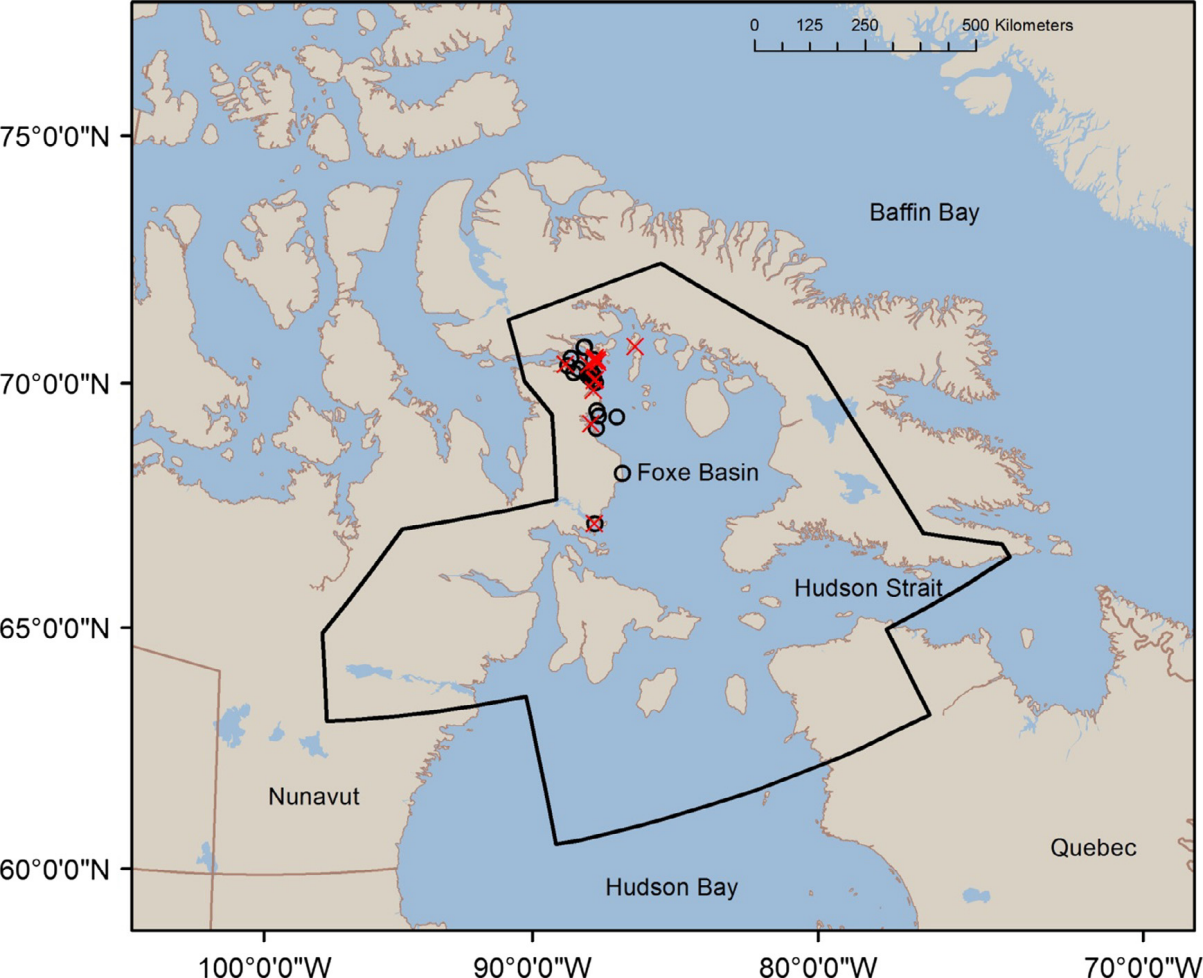
Location of killer whale attacks on bowhead whales (*n* = 13) and bowhead whale carcasses (data from interviews in Ferguson et al. 2012a). Locations of killer whale attacks are represented as (x) and bowhead whale carcasses are represented as (**o**). The Foxe Basin polar bear subpopulation is indicated by the solid line (Obbard et al. 2010).

Historically, killer whales were absent in Hudson Bay and Foxe Basin due to heavy pack ice in Hudson Strait which limited their migration from Davis Strait (Higdon and Ferguson 2009). However, the range of killer whales began expanding into Hudson Bay and Foxe Basin in the 1950s and continued concurrently with sea ice declines in Hudson Strait. Killer whales are now annually present in the region (Higdon and Ferguson 2009; Higdon et al. 2012), which has likely altered local food web dynamics as killer whales and polar bears may depredate the same species. However, killer whale predation may also supplement polar bear diets by providing bowhead whale carcasses, which may be most likely to wash ashore during the open‐water period, when other marine mammal prey are largely unavailable. Hunters have reported killer whale attacks and/or dead bowhead whales throughout the Foxe Basin polar bear subpopulation; however reports are most common in northern Foxe Basin (Fig. 5; data from interviews conducted by Ferguson et al. 2012a). Carcasses are usually attributed to killer whale attacks based on external condition, such as bite marks, chunks of flesh removed, and evidence of internal injury (NWMB 2000, Ferguson et al. 2012a). The tendency for killer whales to target smaller whales such as calves and juveniles, which are found in the highest densities at the northern end of the study area (Ferguson et al. 2012b), is consistent with the highest levels of bowhead consumption among polar bears in northern Foxe Basin (Fig. 2). Furthermore, a greater abundance of bowhead whales was estimated in northern Foxe Basin (e.g., 2760 whales, 95% HDI 1980–5050 in Igloolik) compared to southern Foxe Basin (e.g., 38 whales, 95% HDI 20–124 in Repulse Bay; Frasier et al. 2015), again consistent with the higher levels of bowhead whales found in the diets of northern Foxe Basin polar bears. With evidence of the increased presence of killer whales in Foxe Basin (Higdon et al. 2014) and a growing bowhead whale population (Frasier et al. 2015), it is likely that scavenging opportunities for polar bears will increase over time in Foxe Basin.

### Age‐ and sex‐specific variation in polar bear diet

Age‐ and sex‐specific foraging patterns in Foxe Basin were consistent with adult male bears having the most diverse diets and the greatest ability to capture large prey (Fig. 3). Adult male polar bears may be twice as large as adult females (Derocher et al. 2005, 2010) and are thus better equipped to capture and subdue bearded seals and walruses, which may reach adult body masses in excess of 300 kg and 1000 kg, respectively (Kastelein 2002; Kovacs 2002). Adult female and subadult polar bears in Foxe Basin relied more heavily on ringed seal prey (Fig. 3), and may consume larger prey by scavenging the remains of kills made by adult males (Stirling and McEwan 1975; Derocher et al. 2002). Walrus consumption was highest and most frequent among adult male polar bears, suggesting that these prey are actively hunted, rather than scavenged. The near‐zero consumption of beluga whale by polar bears in this study suggests that belugas are largely unavailable, either as prey or carrion, to polar bears in Foxe Basin, and is consistent with evidence from Ferguson et al. (2010) that killer whale predation on beluga whale is rare in Foxe Basin.

Scavenging of bowhead whale was highest and most frequent among subadult bears, which suggests bowheads may be especially important for less experienced polar bears, and are not defended by potentially despotic adult males. Our finding that bowhead was present in the diets of all age classes and sexes is consistent with observations from the Beaufort Sea that a large whale carcass will attract a large number of bears which subsequently share the resource in the absence of aggressive competitive interactions (Schliebe et al. 2008; Herreman and Peacock 2013; Miller et al. 2015).

### Seasonal variation in polar bear diet

Seasonal variation in the diets of polar bears in Foxe Basin may reflect changes in prey availability associated with seasonal sea ice conditions. Fatty acid profiles reflect integrated diet composition over the preceding weeks and months (Iverson et al. 2004) and peak ringed seal availability is thought to occur during pupping and molting in spring and summer (i.e., April–July; Stirling and Øritsland 1995). Thus, the strongest signal of ringed seal consumption would be expected in the summer. Our finding that ringed seal consumption was highest during fall in northern Foxe Basin was likely a consequence of our lack of summer samples.

Polar bears showed a seasonal increase in walrus consumption in all three regions during early spring. Although walrus are available year‐round within the three areas, the species may become particularly vulnerable to predation during late winter and early spring if heavy ice conditions limit access to open water, leaving walruses potentially stranded on the ice (Calvert and Stirling 1990; DFO 2002). Polar bears may also have access to walrus carcasses after stampeding events at large haul‐outs, however this type of mortality would be limited to the open‐water season (Kochnev 2002). In Hudson Strait (i.e., near the Foxe Basin subpopulation boundary, Fig. 1), harp seal comprised a larger portion of polar bear diets in the spring (46%) than the winter (25%), which may reflect increased vulnerability and accessibility of harp seals and particularly their newborn pups during the whelping period (March) off Newfoundland and Labrador (Stirling and Parkinson 2006; DFO 2011).

The effects of climate change on polar bears has been most extensively studied in the Western Hudson Bay and Southern Beaufort Sea subpopulations, with evidence of individual‐ and population‐level effects including reduced body condition, reproduction, survival, and abundance associated with declining sea ice (Stirling et al. 1999; Regehr et al. 2007; Hochheim et al. 2010; Bromaghin et al. 2015). Polar bears in Baffin Bay, Davis Strait, and Southern Hudson Bay, which are also seasonal sea ice ecoregions (Amstrup et al. 2008), have also shown signs of reduced body condition in relation to sea ice declines (Rode et al. 2012; Obbard et al. 2016). Foxe Basin has a seasonal ice regime and polar bears have experienced an earlier sea ice breakup and later freeze‐up, similar to trends in Western Hudson Bay (Regehr et al. 2007), Baffin Bay, and Davis Strait (Stirling and Parkinson 2006), however population size has remained stable (Obbard et al. 2010; Stapleton et al. 2016). Although continued habitat deterioration will ultimately lead to reduced body condition and cub production in Foxe Basin, the recent lack of demographic response to habitat decline can provide insight into broader ecological processes.

Region‐specific patterns in both diet composition (this study) and space‐use (Sahanatien et al. 2015) suggest that ongoing sea ice declines, and polar bear responses to those declines, are regionally variable. With the longest on‐ice period, smallest home ranges (Sahanatien et al. 2015), and greatest access to bowhead whale, bears in northern Foxe Basin may be least vulnerable to near‐term habitat declines. In contrast, bears in southern Foxe Basin have the shortest on‐ice period, largest home ranges, and least diverse diet, and thus may be most sensitive to warming‐related declines in sea ice. Local and regional responses to ongoing habitat and food web changes could have substantial effects on population vital rates and thus should be incorporated into polar bear conservation and management plans (Thiemann et al. 2008).

Across the Foxe Basin subpopulation of polar bears, the relatively wide diversity of prey and the ability of some bears to shift between locally or seasonally available foods may help mitigate some of the negative effects of habitat loss, at least in the near term (Rode et al. 2014). Periodic availability of multiyear ice during the summer in Foxe Basin may provide occasional hunting opportunities during summer months (Sahanatien and Derocher 2012). Bowhead whale carcasses may represent an increasingly important food resource during the ice‐free period if killer whale predation continues to increase (Reinhart et al. 2013), but the availability of ice‐associated bowheads may also decline with continued deterioration of sea ice. At present, the consumption of bowhead whale, especially by subadult bears (Fig. 3B), may contribute to the apparent demographic stability of the Foxe Basin subpopulation. In other polar bear subpopulations, subadult survival has been particularly sensitive to declines in habitat quality (e.g., Regehr et al. 2007; Bromaghin et al. 2015). Further analysis of long‐term changes in polar bear diets, especially bowhead whale consumption, as a consequence of sea ice decline and killer whale range expansion, would provide important insights into a potential ecological regime shift happening in Foxe Basin.

## Conflict of Interest

None declared.

## Supporting information


**Appendix S1.** Diet simulation.
