# Supplementary material for: Dietary habits of polar bears in Foxe Basin, Canada: possible evidence of a trophic regime shift mediated by a new top predator
Source: Ecol Evol. 2016 Jul 28;6(16):6005–18. doi: 10.1002/ece3.2173 (PMC4983609; doi:10.1002/ece3.2173)
Supplement: Supplementary file 1 — Appendix S1. Diet simulation. [file ECE3-6-6005-s001.docx]

# Appendix A

## Diet Simulation

Diet simulations were performed using all potential prey species geographically available to polar bears in Foxe Basin. The diet simulations were conducted according to Iverson *et al.* (2004), where a subset of prey data were used to create a “pseudo bear” with a hypothetical diet (for instance, 70% ringed seal and 30% bearded seal). The remaining prey data were used as a modelling set to estimate the diet of the pseudo bear. Simulations were repeated 1000 times (Iverson *et al.* 2004). Although previous studies have found within-species variation in prey FA profiles (Thiemann, Iverson & Stirling 2008), this variability is generally not large enough to resolve in QFASA diet estimates. Thus, separating prey by sex or age class generated less accurate diet estimates in simulations. With prey types separated by species, ringed seals and bearded seals were slightly underestimated, whereas harp seal was slightly overestimated (Fig. A).


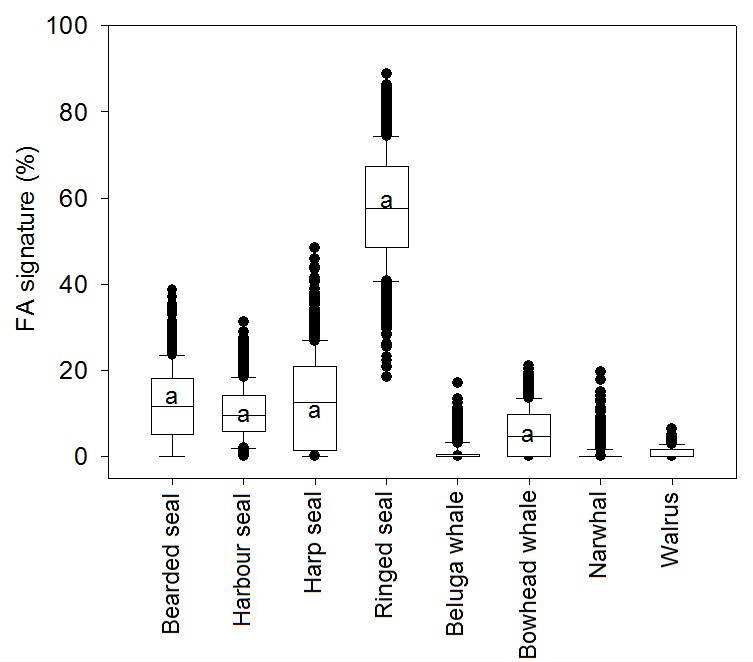


Fig. A. Diet simulation results for the Foxe Basin subpopulation prey dataset which are presented as boxplots showing the 25^th^, median and 75^th^ percentiles of the 1000 diet estimates and solid circles represent outliers. The simulated diet composition is represented as ‘a’ in plots and designated as followed: 15% bearded seal, 0% beluga whale, 5% bowhead whale, 10% harbour seal, 10% harp seal, 0% narwhal, 60% ringed seal, and 0% walrus.
